# Cross-sectional study on nurses’ attitudes regarding coercive measures: the importance of socio-demographic characteristics, job satisfaction, and strategies for coping with stress

**DOI:** 10.1186/s12888-018-1756-1

**Published:** 2018-06-04

**Authors:** Branko Bregar, Brigita Skela-Savič, Blanka Kores Plesničar

**Affiliations:** 1 University Psychiatric Hospital Ljubljana, Studenec 48, 1260 Ljubljana Polje, Slovenia; 2 Angela Boškin Faculty of Health Care, Ljubljana Polje, Slovenia

**Keywords:** Nurse, Restraint, Seclusion, Psychiatry

## Abstract

**Background:**

Coercive measures are containment methods used in psychiatry to curb patients’ disruptive and aggressive behaviours towards themselves, others or objects. The prevalence of the practice of coercive measures in psychiatry is directly related to the attitudes of the staff. When discussing these attitudes, nurses are often particularly singled out. The purpose of the study is to research the impact of individual factors on nurses’ attitudes in the decision-making process for the use of coercive measures.

**Methods:**

A cross-sectional study among all psychiatric nursing staff in Slovenia (*n* = 367, 79%) was conducted over the years 2013/2014. Standardized questionnaires were used, including a survey of nurses’ attitudes to the use of seclusion, the Job Descriptive Index, and the Folkman-Lazarus test.

**Results:**

Nurses’ attitudes towards special coercive measures are predominantly negative ($\overline{x}$ = 11.312, SD = 2.641). The factors that explain a positive attitude are as follows: female gender (β = − 0.236, *p* <  0.001), fewer years of service (β = − 0.149, *p* = 0.023), emotion-focused strategies of coping with stress (β = 0.139, *p* = 0.020), and less-threatening patient behaviour (β = 0.157, *p* = 0.012).

**Conclusions:**

The effects of some known factors did not prove important in the model. Newly recognized factors are “less-threatening patient behaviour” and “emotion-focused strategies of coping with stress”. Therefore, attitudes towards special coercive measures in psychiatry must be regarded as contextualized, interactive, and multidimensional phenomena that cannot be explained merely through a defined set of factors.

## Background

Coercive or containment measures in medicine, especially in psychiatry, are regarded as a serious violation of an individual’s rights to self-determination and to personal freedom [1]. These measures impinge upon the freedom, autonomy and/or movement of hospitalized patients. Manual or physical, mechanical, and chemical restraint and seclusion are used most often in psychiatric institutions across Europe [2–5]. Although all coercive measures (CM) in psychiatry are considered detrimental to the patient’s physical and mental health or to their basic human rights, differences in the legislation, definition, and incidence of CM are still profound between individual European countries and beyond, rendering the comparison between countries impossible to research and study. Therefore, while the consequences of CM are well researched, there is still a need for common definitions and clinical practices [5–9]. In Slovenia, for instance, only mechanical restraint with five-point belt restraints is permitted in psychiatric hospitals according to the Slovenian Mental Health Act of 2008 [10].

Many studies in the past 10–15 years have identified a series of factors that influence the application of coercive measures by staff [11–16]. The most common such factors are the following: the staff’s gender, having a non-stimulative therapeutic relationship with the patient, the staff’s experiences and skills, therapeutic (non-) communication, the relationship between the staff and the patient, the presence of staff among the patients, the personal characteristics of the staff, and other factors [11, 12, 17, 18].

However, despite this seemingly scholarly consensus regarding these factors, there are a number of studies that call into question the relevance of certain individual ideas to the study of CM. For instance, Boumans et al. did not establish a connection between gender and the incidence of CM [19]. Beghi et al., on the other hand, argued that male staff and those who are highly qualified are more prone to use coercive measures [17, 20, 21]. As a third stance, Gelkopf et al. criticized the data, suggesting the predominant use of CM among males as relevant to the incidence of CM because it is common practice in clinics for women to call in their male colleagues to cope with violent patients [18]. The number of years of experience the staff has is another factor that should be carefully considered [18, 22]. The influence of individual socio-demographic factors on CM is thus quite complex, preventing the possibility of a consensus among practitioners and researchers.

In addition, some authors also underscore the fact that nurses, due to the nature of their work, are also usually the first to decide whether to implement CM as a response to a patient’s disruptive behaviour [23]. However, the decision-making process is very complex in every clinical environment because nurses cannot implement CM without the approval of a physician, who has the final authority regarding whether the evaluation of the patient’s behaviour justifies the use of CM.

Within the setting of clinical practice, CM pose an unpleasant and stressful situation for staff [24–26], who develop a variety of coping strategies to address this stress. Such strategies are either emotion-focused or problem-focused [27]. In the second instance, staff actively cope with their stress resulting from CM (problem-focused coping), while passive coping (emotion-focused coping) is employed in the former coping strategy. Active coping is dominant when a person believes a situation can be resolved or changed and when they have the required resources (e.g. knowledge). Passive coping is constructive when facing a final and unchangeable event (e.g., death), but it often works like avoidance and/or denial, which are not constructive [27]. This means that an individual would rather avoid stressful situations than cope with them. The importance of coping with stress among nurses has been studied by other researchers, though all of them worked outside the scope of CM. Gholamzadeh et al. discovered that large numbers of nurses tend to use passive coping [28]. Emotion-focused strategies for coping with stress are more common among female nurses with a long period of employment and among those with lower levels of education [29].

Research regarding strategies for coping with stress as a result of CM is justified not only by actual studies on the strategies used for coping with stress in other fields but also by studies such as those by Happel and Koehn, indicating that the higher the nurse’s level of job satisfaction, the higher the quality of their work performance [30, 31]. More importantly, if nurses are more satisfied with their jobs, they are more likely to have a negative attitude towards CM, which itself is the most frequently identified cause of stress, thereby reducing the likelihood that nurses resort to CM.

The impact of coping with stress on CM has not yet been studied (per se). Since some studies show an impact as well as contradictions between factors influencing nurses’ attitudes towards CM, we believe that these factors must be contextually studied in each environment individually.

## Methods

### Aim

The goal of this study was to research the influence of gender, age, years of service, education, working environment (closed/open ward), differences per individual psychiatric hospital (5 regional hospitals and one clinic), and job satisfaction, as well as the various strategies for coping with stress, on such attitudes among nurse practitioners in Slovenia. The goal was to consider the relevant factors when seeking to reduce CM.

### Design

A cross-sectional descriptive study was conducted using a structured questionnaire that was completed by the nursing staff in all the psychiatric hospitals in Slovenia.

### Participant characteristics

All the psychiatric hospitals (*n* = 6) were included in the sample. The study included the entire population of psychiatric nursing staff active on a certain day. According to the data from 2013 and 2014, six psychiatric hospitals employed 464 people in nursing care, of whom 118 were graduated nurses and 346 were health care assistants; of these, there were 367 nurses (79%) who participated in the study. Their mean age was 38.04 (SD = 9.490), and their mean years of service was 17.10 (SD = 9.940). The majority of research respondents, namely, 71.8% (*n* = 264), had secondary school-level medical education (health care assistant), while the remainder had a bachelor’s degree in nursing (registered nurse) or were post-graduate educated. Most (*n* = 255, 69.6%) of the respondents had already done work in a closed psychiatric ward.

### Instrument

The instrument consisted of four sets of questions. The first set’s goal was to collect the following socio-demographic data: gender, age, education, years of service in a mental health setting, workplace (closed/open ward of a psychiatric hospital) and hospital.

The second set consisted of the Heyman questionnaire on personnel’s attitude towards CM – survey of nurses’ attitudes to seclusion survey (SNASS) [32]. This questionnaire does not have sub-scales. Every statement in the analysis has been used as a separate variable [30]. In our analysis, we considered two sets related to our research: 1) the reasons and justification for implementing CM – every statement requires an answer: never, sometimes, and often; and 2) employees’ feelings related to CM – every statement requires an answer: never, sometimes, and often.

The third set used the Job Descriptive Index (JDI) questionnaire, which measures job satisfaction [33] through 106 statements. The analysis of the answers to individual statements (1 – yes, 2 – no, 3 – undefined; every answer has its weight) facilitates the evaluation of five dimensions of job satisfaction: 1) job satisfaction per se, 2) management satisfaction (direct manager), 3) co-worker satisfaction, 4) salary satisfaction, and 5) promotion possibility satisfaction. The final result is an aggregate of all five dimensions or is an evaluation of the individual dimensions.

The fourth instrument set was intended to evaluate stress coping strategies. The Folkman-Lazarus test was used – Ways of Coping Questionnaire (WCQ) – which enables insight into processes or strategies of coping with stress [34]. The questionnaire consists of 66 statements, measured on a scale from 0 (not at all) to 3 (somewhat), which is used to measure 8 dimensions of coping with stress: confrontation, distancing, self-control, seeking social help, accepting responsibility, withdrawal/avoidance, planned problem solving, and positive re-evaluation.

### Validity and reliability

#### SNASS

To implement the questionnaire in the Slovenian language, we followed the rules for translation and usage. Reliability was between 0.58 and 0.80 of Cronbach’s alpha: 1) reasons and justification for CM implementation, 13 statements (α = 0.80); and 2) employee’s feelings, related to CM, 13 statements (α = 0.58). When the statement “satisfaction with helping patients” was removed, the sum of Cronbach’s alpha yielded α = 0.64. The removed statement was not used in the ensuing statistical processes. The authors who used SNASS in their research evaluated every statement in the analysis separately; however, they did not try to reduce the statements in the individual sets [30, 31]. In our next step, we analysed the following factors:

1. Reasons for implementing CM – considering the structure of statements, we wanted to determine whether all 13 statements denoting the reasons for CM in the questionnaire could be combined into a smaller number. The analysis of the main components showed that the data are suitable for a factor analysis (correlation matrix shows coefficient values > 0.3; KMO = 0.830 and Bartlett’s test of Sphericity *p* <  0.001) [35]. The results also indicated that three components or factors had values exceeding 1, which explained 56.13% of the common variance. However, using the results of the Monte Carlo parallel analysis, we extrapolated that keeping two components is a reasonable approach [35], prompting us to orthogonally varimax-rotate the data with two fixed factors. This explains 48.37% of the common variance, with the first factor and second factor explaining 29.15 and 19.19%, respectively. The first factor also classified the statement “the patient is becoming excited and out of control”, which, despite having a weight under 0.5, it in substance belongs with the factor of “less-threatening patient behaviour” (α = 0.826). The second factor was “threatening patient behaviour” based on the largest weight of the individual statements (α = 0.721). We excluded “the patient is asking to be restrained” (this statement has a very low weight) (Table 1).
2. Regarding the employee’s feelings related to CM, since feelings are linked to attitudes towards a certain situation or person, these statements were dedicated to further analysis. The data lent themselves to reduction (correlational coefficient values > 0.3; KMO = 0.762 and Bartlett’s test of Sphericity *p* <  0.000) [35]. The analysis of the main components indicated that three components can explain 54.43% of the common variance. However, using a parallel test makes it sensible to use two factors [35]. Orthogonal varimax rotation with two fixed factors was performed, resulting in two factors that explain 43.76% of the common variance, with the first and second factor explaining 28.23 and 15.52%, respectively. Individual variables were arranged with strong weights to individual factors, except “fed up” and “powerful”, which were excluded (both statements had weights of less than 0.3). With respect to the highest weight of the individual factors, the first one was identified as “negative attitude towards CM”, which indicated that the staff has difficulties deciding about CM or have prejudices towards CM implementation (α = 0.81). The second factor was identified as “positive attitude towards CM”, which indicated that the staff easily decides to implement CM (α = 0.67) (Table 1).

**Table 1:** Descriptive statistics results for reasons for the CM use and statements for nurses’ emotional responses and factor analysis results

Statements about the reasons for the CM	n	$\overline{x}$	SD	F 1*	F 2**	Statements for nurses’ emotional responses	n	$\overline{x}$	SD	F 1***	F 2****
The patient is becoming excited and out of control	338	2.23	0.588	**0.406**	0.208	Annoyed that the patient was secluded	343	2.02	0.646	**0.602**	−0.082
The patient is hitting another patient	337	2.46	0.566	−0.093	**0.825**	Relieved that the problem has been resolved	341	2.30	0.608	−0.120	**0.730**
The patient is yelling and making too much noise	339	1.68	0.643	**0.724**	0.094	Satisfied that the ward is running smoothly	340	2.15	0.698	−0.115	**0.795**
The patient is hitting a staff member	336	2.48	0.588	−0.074	**0.770**	Guilt or misgivings about the necessity for secluding the patient	338	1.65	0.568	**0.748**	−0.060
The patient wants to sleep	340	1.14	0.381	**0.590**	0.003	Regretful that the crisis was not resolved differently	345	1.91	0.638	**0.671**	0.072
The patient is showing inappropriate sexual behavior	340	1.60	0.608	**0.582**	0.367	Powerful	345	1.06	0.228	0.051	0.295
The patient is annoying or interrupting other people	336	1.49	0.608	**0.772**	0.042	Angry that it was a mistake to have secluded the patient	342	1.28	0.451	**0.687**	−0.041
The patient is trying to break something like a chair or window	338	2.51	0.562	0.167	**0.682**	That you have failed	342	1.56	0.521	**0.735**	−0.003
The patient is cursing or swearing at other people	337	1.50	0.618	**0.831**	0.014	Disempowered that it ended this way	340	1.63	0.553	**0.758**	−0.060
The patient is trying to hurt him/herself	337	2.57	0.558	0.141	**0.621**	In control of the situation	341	1.80	0.665	−0.070	**0.718**
The patient will not take his/her medication	336	1.42	0.646	**0.771**	−0.082	Fed up	341	1.26	0.469	0.263	0.280
The patient is waking other patients at night	341	1.73	0.615	**0.648**	0.234	Angry at being made to be involved in a process that I do not agree with	341	1.31	0.501	**0.572**	0.018
The patient is asking to be restrained	342	1.87	0.604	0.228	0.350	/	/	/	/	/	/

*Legend:* $\overline{x},$ mean; n, number of responses; SD, standard deviation; F1*, less-threatening behaviour; F2**, threatening behaviour; F*** negative attitude towards CM; F**** positive attitude towards CM; significance of bold values at the 0.05 level

#### JDI

The JDI questionnaire proved to be a reliable instrument with validation (α > 0.7) in all five dimensions [36]. The reliability of the entire questionnaire in our case was α = 0.71.

#### WCQ

The internal consistency of the questionnaire in individual dimensions of coping with stress ranged between 0.61 and 0.79 [34]. The reliability of the entire questionnaire was 0.92 in our case. Since the WCQ test was developed to evaluate coping with stress in two directions, namely, problem-focused coping strategies and emotion-focused coping strategies [37], we proceeded with the following analysis to perform a factor analysis with the fixed factors previously discussed. The data the facilitated factor analysis (correlational coefficient > 0.3, KMO = 0.884, Bartlett’s test of Sphericity *p* <  0.001). The analysis of the main components demonstrated the sensibility of keeping two components, which together explained 61.07% of the common variance, with the first and second component explaining 47.03 and 14.05%, respectively. Both components had values greater than 1. We then performed orthogonal varimax rotation with two fixed factors. Both factors explained 61.08% of the common variance, with the first and second explaining 34.07 and 26.99%, respectively. The first factor, “problem-focused coping strategy”, consisted of the following dimensions: planned problem solving, positive re-evaluation, seeking social help, and accepting responsibility (α = 0.761). The other factor, “emotion-focused coping strategy”, comprised the following: withdrawal/avoidance, distancing, self-control, and confrontation (α = 0.715) (Table 2). However, by clustering “accepting responsibility” and “confrontation” factors, we decided to determine them within the theoretical framework instead of within the weight (26).

**Table 2:** Descriptive statistics of the WCQ test and factor analysis

WCQ test	n	$\overline{\mathbf{x}}$	SD	F 1	F 2
Planned problem solving	362	7.268	2.69	**0.855**	0.085
Positive re-evaluation	364	6.318	2.588	**0.815**	0.146
Seeking social help	362	10.107	3.173	**0.663**	0.245
Confrontation	363	8.443	3.186	0.517	**0.447**
Withdrawal/avoidance	362	6.309	1.795	− 0.35	**0.895**
Distancing	355	4.659	3.634	0.281	**0.708**
Self-control	361	11.653	2.876	0.525	**0.544**
Accepting responsibility	361	10.620	3.377	**0.519**	0.526

*Legend: n, number of responses;* $\overline{x},$ *mean; SD, standard deviation; F1, problem-focused coping strategy; F2, emotion-focused coping strategy. Significance of the bold values is at the 0.05 level*

### Description of the research course and data processing

In each of the six Slovenian psychiatric hospitals that we examined, we found a key person to help us conduct our research. We briefly explained the purpose of our study and how to distribute and collect the questionnaires. The study was conducted during the years 2013/2014. The respondents needed approximately 20 min to complete the questionnaire. Our research method defined CM as a physical restraint, a mechanical restraint (use of belts to fix a patient to a bed), or a seclusion, according to the Slovenian Mental Health Act (Official Gazette of the RS nr.77/2008). According to the Mental Health Act, only physicians can make the decision to apply CM, though the decision is often made based on the advice of nurses.

The data were analysed with the statistical analysis software SPSS 22 (IBM Corp. Released 2013. IBM SPSS Statistics for Windows, Version 22.0. Armonk, NY: IBM Corp.) and G*Power for computing statistical power [38, 39]. We used the basic descriptive analysis, the non-parametric Mann-Whitney test, and the Kruskal-Wallis test, as well as Spearman’s correlation coefficient and linear regression. For all the scales where a factor analysis was conducted, we employed a principal axis factoring approach to the factor analysis (rotation method: varimax), the Bartlett sphericity test (*p* < 0.05), and the KMO measure (> 0.6) [35]. A regression analysis was used to study the positive attitudes towards CM, which is problematic and requires careful examination in every clinical field. In the regression, positive attitude was the independent variable, and the following dependent variables, which proved to be correlated with the independent variable (*ρ* > 0.10, *p* < 0,05), were used: gender, education, age, years of service, ward, hospital, emotion-focused coping strategies, less-threatening patient behaviour, and threatening behaviour. Age was excluded in the following step, as the collinear statistical values in the years of service (tolerance = 0.054, VIF = 18.400) and age (tolerance = 0.053, VIF = 18.799) were high. In such cases, Pallant advises the exclusion of one of the variables. Since both variables can be interpreted similarly, we decided to retain “years of service” since it is more relevant to our study [35]. Statistical significance was set at the *p* < 0.05 level.

The study design with the oral consent of the participants was approved by the National Medical Ethics Committee of Slovenia (decision No. 37/0315, 2015).

## Results

### SNASS – Reasons for CM

The descriptive analysis of the first SNASS set, which combined 13 statements into two factors explaining the reasons to implement CM, showed that respondents tended to decide in favour of CM more often in cases of less-threatening patient behaviour ($\overline{x}$= 12.782, SD = 3.194). Men, compared to women, were more likely to decide to use CM in the case of threatening (U = 9837.00, *p* = 0.005) or less-threatening (U = 9666.50, *p* = 0.023) patient behaviour, while those working in closed wards believed that threatening patient behaviour was more often a reason to implement CM (U = 9503.50, *p* = 0.014) (Table 3).

**Table 3:** Forms of behaviour and attitudes towards CM according to gender, education, ward (open/closed) and hospitals

Behavior	n	$\overline{\boldsymbol{x}}$	SD	Gender (U/p)	Education (U/p)	Ward (U/p)	Hospital (χ^2^(2)/p)	Attitude	n	$\overline{\boldsymbol{x}}$	SD	Gender (U/p)	Education (U/p)	Ward (U/p)	Hospital (χ^2^(2)/p)
Less-threatening behavior	317	12.782	3.194	9666.50 0.023	9306.00 0.133	10,432.50 0.529	19.913 0.001	Positive	336	6.247	1.547	8684.50 < 0.001	9612.50 0.016	9556.50 0.010	17.666 0.003
Threatening behavior	327	10.048	1.664	9837.00 0.005	10,456.00 0.408	9503.50 0.014	13.264 0.021	Negative	326	11.312	2.641	10,961.00 0.150	9961.00 0.253	10,264.00 0.276	23.371 < 0.001

*Legend: n, number of responses;* $\overline{x\ }$ *, mean; SD, standard deviation; U, Mann-Whitney test; χ* ^*2*^ *(2), Kruskal-Wallis test; p, statistical significance*

### SNASS – Employees’ feelings related to CM

The scale of 12 statements addressing the question “What do you usually feel after CM?” was combined into two factors using a factor analysis: 1) positive attitude towards CM and 2) negative attitude towards CM. Table 3 shows the basic descriptive statistics of both factors. Male respondents (U = 8684.50, *p* < 0.001) with lower education (U = 9612.50, *p* = 0.016), as well as those who worked in closed wards (compared to those who work in open wards) (U = 9556.50, *p* = 0.10), were significantly different in their favouring of CM (Table 3).

### JDI – Descriptive statistics

The respondents were the most satisfied management ($\overline{x}$ = 64.243, SD = 15.491) and their co-workers ($\overline{x}$ = 60.454, SD = 13.837), job ($\overline{x}$ = 14.297, SD = 11.101), and personal income ($\overline{x}$ = 11.806, SD = 8.716), and they were least satisfied with their promotion possibility ($\overline{x}$ = 10.214, SD = 7.920). Satisfaction in all five dimensions together did not show any significant differences for each gender (U = 7947.50, *p* = 0.289), open/closed ward (U = 7704.50, *p* = 0.387), or hospital (χ2(2) = 10.331, *p* = 0.066). In regard to education, those with higher education were significantly different in the satisfaction with their work (U = 5629.00, *p* < 0.000).

### WCQ – Descriptive statistics

The results showed that the respondents addressing stressful situations more likely turn to problem solving ($\overline{x}$ = 37.104, SD = 8.801) than to emotions ($\overline{x}$ = 28.387, SD = 8.978). Individual dimensions of stress coping strategies with respect to gender, open/closed ward, or hospital did not show any important significant differences. In regard to education, those with higher education turned to problem-solving strategies more often than those with lower education, with a significant difference (U = 10,146.00, *p* = 0.003) (Table 4).

**Table 4:** Descriptive statistics of the WCQ according to gender, education, ward (open/closed) and hospital

Coping strategy	n	$\overline{\boldsymbol{x}}$	SD	Gender (U/p)	Education (U/p)	Ward (U/p)	Hospital (χ^2^(2)/p)
Problem-focused coping strategies	355	37.104	8.801	13,566.00 0.561	10,146.00 0.003	12,769.50 0.430	6.607 0.252
Emotion-focused coping strategies	351	28.387	8.978	13,639.00 0.906	11,779.00 0.368	12,140.00 0.296	5.196 0.392

*Legend: n, number of responses;* $\overline{x\ }$ *, mean; SD, standard deviation; U, Mann-Whitney test; χ2(2), Kruskal-Wallis test; p, statistical significance*

### Regression model

The final model explained 21.9% of the variance in positive attitudes towards CM. The important significantly different independent variables that explained the model include the following: female gender, a low number of years of service, emotion-focused coping strategies, and less-threatening patient behaviour. The female gender variable had the strongest explanatory power (Table 5).

**Table 5:** Regression model for positive attitudes

Positive attitudes to CM (R_adj._^2^ = 0.218, *p* < 0.001)				
Independent variables	b	SE	β	p
Gender	−0.738	0.199	−0.236	**< 0.001**
Education	−0.112	0.206	− 0.034	0.587
Years of service	−0.024	0.011	−0.149	**0.023**
Ward	0.080	0.212	0.025	0.705
Hospital	−0.018	0.054	−0.019	0.745
Emotion-focused coping strategies	0.023	0.010	0.139	**0.020**
Less-threatening behaviour	0.073	0.029	0.157	**0.012**
Threatening behaviour	0.067	0.056	0.073	0.230
JDI satisfaction with work	−0.016	0.009	−0.118	0.080
JDI satisfaction with salary	0.006	0.012	0.036	0.597

*Legend: R2, R-Squared; b, regression coefficient; SE, standard error of regression coefficient; β, standard regression coefficient; p, statistical significance*

The statistical power of the computed multiple linear regression resulted as 1, which is higher than 80% as recommended by the literature.

## Discussion

Our study that aimed to explain nurses’ attitudes towards CM not only corroborates some of the known factors such as gender and years of service but also highlights new factors, in particular, less-threatening patient behaviour and emotion-focused coping strategies. Nonetheless, while the results of the present study on Slovenian nurses generally overlap with the results of similar studies abroad, some of the findings are in conflict with the findings and trends of other studies. For instance, most studies show that the lower education of nursing staff and working in closed psychiatric wards are correlated with a more positive attitude towards CM [8, 40]. We drew the same conclusion at first, when these factors were correlated separately, but once the data were analysed in a regression model, they did not show a meaningful impact on the nurses’ attitudes towards CM. This result explains why the factors influencing nurses’ attitudes towards CM should not be investigated separately but rather jointly. This observation concurs with several recent studies that stress that causes of CM must primarily be sought within the organization and culture of individual health institutions and not at the level of the clinical characteristics of a patient or the staff [41, 42]. Further, we expected to find a correlation between the nurses’ attitude towards CM and job satisfaction, as Happell et al. established a weak correlation between job satisfaction and attitudes towards seclusion [23]. However, we were unable to confirm this correlation, most likely because the methodological blueprint of researching the correlation between job satisfaction and attitudes towards CM requires further improvement, as also noticed by Happell et al. [23], and because job satisfaction is indirectly linked to the results of health treatment [43].

Another divergent finding of our study relates to gender and years of service, i.e., work experience. Gender and years of service – or work experience – are well-known factors [40]. Contrary to Happel & Harrow, who found that males are more inclined to use CM, our research revealed that women have a more positive attitude towards CM. Many studies have argued for the predominant influence of the male gender on the prevalence of CM, as men are usually those who implement CM [11, 17, 20]. However, these studies largely ignore the procedural nature of CM; for instance, women are usually the ones who decide much earlier and require help for the implementation of CM. Therefore, in our opinion, we cannot assess gender as a factor influencing CM simply through the prism of the quantitative results.

One of the important factors for implementing CM in our study proved to be patients’ threatening behaviour (defined as violence towards other people, inventory or themselves). Implementing CM could have a positive effect in decreasing frequencies of violence towards Slovenian nurses, as found in some Slovenian researches [44–47]. This finding corresponds with the findings of previous research, where such behaviour is identified as one of the most common reasons for the implementation of CM [11, 17, 18, 20, 23, 30, 48–53]. However, when we analysed different factors in the regression model, patients’ threatening behaviour did not prove to be statistically significant. Instead, patients’ “less-threatening behaviour” prevailed. The respondents in our study perceived patients’ “less-threatening behaviour” (such as uncontrolled excitement of a patient, screams and noise, inappropriate sexual behaviour, etc. (see Table 1) as a more justified reason for the use of CM than the genuinely “threatening behaviour”, even if CM in such cases was unwarranted from both a professional and legal perspective, according to the Slovenian Mental Health Act. In fact, respondents most often implemented CM in such cases on the basis of their anticipation that the patient’s behaviour would escalate into aggressive behaviour; however, this never occurred due to the CM intervention. This result emphasizes the existence of a “grey area” in the decision-making process of nurses, which was not further examined per se in our study. This “grey area”, however, is not an exclusively Slovenian problem, as several studies from abroad provide similar reports. For instance, Jalil et al. found that nurses show more anger and readiness to use CM if patients insult them, whereas Papadopoulos et al. and Gudde et al. observed that nurses themselves incite violence in patients, which usually results in the implementation of CM [54–56]. Clearly, this issue, which indicates that personal factors such as staff’s ability to cope with frustration also play an important role in the decision-making process, should be addressed and explored more carefully in relation to CM by future studies.

Since nurses most commonly are not equipped with clear instructions or strict guidelines regarding how to address every possible dangerous situation, patient behaviour as well as other adverse events undoubtedly present a constant source of various degrees of stress for nurses, as already established by previous research [57, 58]. To cope with this constant stress, nurses must develop strategies to avoid burn-out and long-term absence from their work place [59].

Our study has shown that a positive attitude towards CM among nurses is linked to emotion-focused strategies for coping with stress, such as withdrawal, avoidance, and distance from problems. This result may comply with the theory proposed by Folkman and Lazarus [27]. On the one hand, we understand that Slovenian nurses are not empowered to change their own clinical practice in this area and must accept the situation as it is since they cannot change it. Another explanation may be that they accept the situation as it is, since it is more comfortable to do so, and they can resign themselves to a daily routine that does not require much effort but may require denial, avoiding confrontation, etc. In both cases, CM are regarded as a static phenomenon that are scripted and defined through protocols and guidelines that cannot be changed through individual experience and do not take into account the individual nature of each case that is involved in the process of implementing coercive measures.

In conclusion, our study explained only a fifth of the cases of nurses’ positive attitudes towards CM, which shows that other influential factors exist. The results show some critical findings that need to be accounted for and improved upon. In addition to the importance of the female gender, which was partially confirmed by other authors, an acute problem lies in working with patients who behave aggressively and insultingly towards nurses. In these cases, nurses must be empowered to take responsibility for changes to the clinical practices in which they work and to learn to utilize problem-focused strategies for coping with stress in critical situations, which means focusing on resolving an actual problem and not deferring to the existing situation.

## Conclusions

A static, decontextualized conceptualization and/or understanding of the process of CM, in our view, poses an underlying obstacle in containing and reducing CM practices in psychiatric settings. Such a conclusion has been reached in our study, which sought to evaluate the attitude of Slovenian nurses with the help of already known factors, as well as some others that were newly proposed for this study. By adopting the perception of CM as a composite and procedural phenomenon, we maintain that management can improve practices by appropriately structuring teams, particularly taking into account the gender of nurses, their years of service, and their knowledge of how to address patient behaviour. On the other hand, nurses need to take responsibility for their work and to make decisions based upon the moral and ethical standards that are valid for all health-care workers.

### Research limitations

The study had a number of methodological weaknesses. The findings implied herein should be researched in studies with stronger methodological conceptions. The study was also limited because not all the respondents answered all the questions; therefore, some data is missing. Because the instruments were translated into Slovenian before their validation, there is the possibility of slight differences in the local terminology. The sample was not balanced according to education, gender, or hospitals/wards, which could also importantly influence the results. International comparisons regarding education cannot be exact because the proportion of nurses with higher education is significantly lower in Slovenia compared to other EU countries. (In psychiatry there is a ratio of 30:70 in favour of health care assistants, which is a secondary education title, but this reflects the actual educational structure of nursing employees in Slovenian hospitals.). Finally, the accuracy of self-report questionnaire techniques may be limited.

## Abbreviations

CM: Coercive measures
JDI: Job Descriptive Index questionnaire
SNASS: Survey of nurses’ attitudes to seclusion survey
WCQ: The Folkman-Lazarus test was used – Ways of Coping Questionnaire

## References

[1] SAMS (2017). Coercive measures in medicine. Bern: house of academies.

[2] Bergk J, Einsiedler B, Flammer E, Steinert T (2011). A randomized controlled comparison of seclusion and mechanical restraint in inpatient settings. Psychiatr Serv.

[3] Nawka A, Kalisova L, Raboch J, Giacco D, Cihal L, Onchev G (2013). Gender differences in coerced patients with schizophrenia. BMC Psychiatry.

[4] Riahi S, Thomson G, Duxbury J (2016). An integrative review exploring decision-making factors influencing mental health nurses in the use of restraint. J Psychiatr Ment Health Nurs.

[5] Steinert T, Lepping P, Bernhardsgrutter R, Conca A, Hatling T, Janssen W (2010). Incidence of seclusion and restraint in psychiatric hospitals: a literature review and survey of international trends. Soc Psychiatry Psychiatr Epidemiol.

[6] Janssen WA, Noorthoorn EO, de Vries WJ, Hutschemeakers GJ, Lendemeijer HH, Widdershoven GA (2008). The use of seclusion in the Netherlands compared to countries in and outside Europe. Int J Law Psychiatry.

[7] Castle NG, Engberg J (2009). The health consequences of using physical restraints in nursing homes. Med Care.

[8] Fariña-López E, Estévez-Guerra GJ, Gandoy-Crego M, Polo-Luque LM, Gómez-Cantorna C, Capezuti EA (2014). Perception of spanish nursing staff on the use of physical restraints. J Nurs Scholarsh.

[9] Steinert T, Noorthoorn EO, Mulder CL (2014). The use of coercive interventions in mental health care in Germany and the Netherlands. A comparison of the developments in two neighboring countries. Front Public Health.

[10] Ivanc B (2008). The Slovenian mental health act de lege ferenda. Med Law.

[11] Cornaggia CM, Beghi M, Pavone F, Barale F (2011). Aggression in psychiatry wards: a systematic review. Psychiatry Res.

[12] Hallett N, Huber JW, Dickens GL (2014). Violence prevention in inpatient psychiatric settings: systematic review of studies about the perceptions of care staff and patients. Aggress Violent Behav.

[13] Doedens P, Maaskant JM, Latour CHM, Meijel B, Koeter MWJ, Storosum JG (2017). Nursing staff factors contributing to seclusion in acute mental health care - an explorative cohort study. Issues Ment Health Nurs..

[14] Husum TL, Bjorngaard JH, Finset A, Ruud T (2010). A cross-sectional prospective study of seclusion, restraint and involuntary medication in acute psychiatric wards: patient, staff and ward characteristics. BMC Health Serv Res.

[15] Lindsey PL (2009). Psychiatric nurses' decision to restrain. J Psychosoc Nurs Ment Health Serv.

[16] Larue C, Dumais A, Ahern E, Bernheim E, Mailhot MP (2009). Factors influencing decisions on seclusion and restraint. J Psychiatr Ment Health Nurs.

[17] Beghi M, Peroni F, Gabola P, Rossetti A, Cornaggia CM (2013). Prevalence and risk factors for the use of restraint in psychiatry: a systematic review. Riv Psichiatr.

[18] Gelkopf M, Roffe Z, Behrbalk P, Melamed Y, Werbloff N, Bleich A (2009). Attitudes, opinions, behaviors, and emotions of the nursing staff toward patient restraint. Issues Ment Health Nurs.

[19] Boumans CE, Egger JI, Souren PM, Mann-Poll PS, Hutschemaekers GJ (2012). Nurses' decision on seclusion: patient characteristics, contextual factors and reflexivity in teams. J Psychiatr Ment Health Nurs.

[20] Bowers L, Van Der Merwe M, Nijman H, Hamilton B, Noorthorn E, Stewart D, Muir-Cochrane E (2010). The practice of seclusion and time-out on English acute psychiatric wards: the City-128 study. Arch Psychiatr Nurs.

[21] Wynn R, Kvalvik AM, Hynnekleiv T (2011). Attitudes to coercion at two Norwegian psychiatric units. Nord J Psychiatry.

[22] Hamers JP, Meyer G, Kopke S, Lindenmann R, Groven R, Huizing AR (2009). Attitudes of Dutch, German and Swiss nursing staff towards physical restraint use in nursing home residents, a cross-sectional study. Int J Nurs Stud.

[23] Happell B, Dares G, Russell A, Cokell S, Platania-Phung C, Gaskin CJ (2012). The relationships between attitudes toward seclusion and levels of burnout, staff satisfaction, and therapeutic optimism in a district health service. Issues Ment Health Nurs..

[24] Moran A, Cocoman A, Scott PA, Matthews A, Staniuliene V, Valimaki M (2009). Restraint and seclusion: a distressing treatment option?. J Psychiatr Ment Health Nurs.

[25] Kuosmanen L, Makkonen P, Lehtila H, Salminen H (2015). Seclusion experienced by mental health professionals. J Psychiatr Ment Health Nurs.

[26] Goulet MH, Larue C, Lemieux AJ (2018). A pilot study of "post-seclusion and/or restraint review" intervention with patients and staff in a mental health setting. Perspect Psychiatr Care.

[27] Folkman S, Lazarus RS, Gruen RJ, DeLongis A (1986). Appraisal, coping, health status, and psychological symptoms. J Pers Soc Psychol.

[28] Gholamzadeh S, Sharif F, Rad FD (2011). Sources of occupational stress and coping strategies among nurses who work in admission and emergency departments of hospitals related to Shiraz University of Medical Sciences. Iran J Nurs Midwifery Res.

[29] Zyga S, Mitrousi S, Alikari V, Sachlas A, Stathoulis J, Fradelos E (2016). Assessing factors that affect coping strategies among nursing personnel. Mater Sociomed.

[30] Happell B, Koehn S (2010). Attitudes to the use of seclusion: has contemporary mental health policy made a difference?. J Clin Nurs.

[31] Happell B, Koehn S (2011). Impacts of seclusion and the seclusion room: exploring the perceptions of mental health nurses in Australia. Arch Psychiatr Nurs.

[32] Heyman E (1987). Seclusion. J Psychosoc Nurs Ment Health Serv.

[33] Smith PC. The measurement of satisfaction in work and retirement: A strategy for the study of attitudes. Chicago: Rand McNally and Company; 1969.

[34] Folkman S, Lazarus RS. Manual for the ways of coping questionnaire: Consulting Psychologists Press; 1988.

[35] Pallant J. SPSS survival manual: a step by step guide to data analysis using SPSS for windows (version 12). 2nd ed. New York: Open University Press, 2005.

[36] Bland RC, Newman SC, Orn H (1997). Age and remission of psychiatric disorders. Can J Psychiatr.

[37] Edwards JR, Baglioni Jr A. Empirical versus theoretical approaches to the measurement of coping: a comparison using the ways of coping questionnaire and the cybernetic coping scale. In: Dewe P, Cox T, Leiter M, editors. Coping and health in organizations. London: Taylor & Francis; 1999 p21-50.38.

[38] Faul F, Erdfelder E, Lang AG, Buchner A (2007). G*power 3: a flexible statistical power analysis program for the social, behavioral, and biomedical sciences. Behav Res Methods.

[39] Faul F, Erdfelder E, Buchner A, Lang AG (2009). Statistical power analyses using G*power 3.1: tests for correlation and regression analyses. Behav Res Methods.

[40] Happell B, Harrow A (2010). Nurses' attitudes to the use of seclusion: a review of the literature. Int J Ment Health Nurs.

[41] Lay B, Nordt C, Rössler W (2011). Variation in use of coercive measures in psychiatric hospitals. Eur Psychiatry.

[42] Di Lorenzo R, Miani F, Formicola V, Ferri P (2014). Clinical and organizational factors related to the reduction of mechanical restraint application in an acute ward: an 8-year retrospective analysis. Clin Pract Epidemiol Ment Health.

[43] Lorber M, Treven S, Mumel D (2015). The importance of monitoring nurses' workplace satisfaction of nurses for the well-being of all employees in nursing. Slov Nurs Rev..

[44] Gabrovec B, Lobnikar B (2015). The analysis of the role of an institution in providing safety and quality in psychiatric health care. Anadolu Psikiyatri Derg.

[45] Gabrovec B, Erzen I (2016). Prevalence of violence towards nursing staff in Slovenian nursing homes. Zdr Varst.

[46] Gabrovec B (2017). Prevalence of violence toward community nurses: a questionnaire survey. Workplace Health Saf.

[47] Gabrovec B, Jelenc M, Prislan K, Lobnikar B (2017). Violence against working personnel in Slovenian drug addiction rehabilitation Centre network. Heroin Addict Relat Clin Probl.

[48] Bai X, Kwok TC, Ip IN, Woo J, Chui MY, Ho FK (2014). Physical restraint use and older patients’ length of hospital stay. Health Psychol Behav Med.

[49] Bullock T, Giesbrecht B (2014). Acute exercise and aerobic fitness influence selective attention during visual search. Front Psychol.

[50] Furre A, Sandvik L, Friis S, Knutzen M, Hanssen-Bauer K (2016). A nationwide study of why and how acute adolescent psychiatric units use restraint. Psychiatry Res.

[51] Cullen AE, Bowers L, Khondoker M, Pettit S, Achilla E, Koeser L (2018). Factors associated with use of psychiatric intensive care and seclusion in adult inpatient mental health services. Epidemiol Psychiatr Sci.

[52] Cowman S, Björkdahl A, Clarke E, Gethin G, Maguire J (2017). A descriptive survey study of violence management and priorities among psychiatric staff in mental health services, across seventeen european countries. BMC Health Serv Res.

[53] Estévez-Guerra GJ, Fariña-López E, Núñez-González E, Gandoy-Crego M, Calvo-Francés F, Capezuti EA (2017). The use of physical restraints in long-term care in Spain: a multi-center cross-sectional study. BMC Geriatr.

[54] Jalil R, Huber JW, Sixsmith J, Dickens GL (2017). Mental health nurses' emotions, exposure to patient aggression, attitudes to and use of coercive measures: cross sectional questionnaire survey. Int J Nurs Stud.

[55] Papadopoulos C, Bowers L, Quirk A, Khanom H (2012). Events preceding changes in conflict and containment rates on acute psychiatric wards. Psychiatr Serv.

[56] Gudde CB, Olso TM, Whittington R, Vatne S (2015). Service users' experiences and views of aggressive situations in mental health care: a systematic review and thematic synthesis of qualitative studies. J Multidiscip Healthc.

[57] Schablon A, Zeh A, Wendeler D, Peters C, Wohlert C, Harling M (2012). Frequency and consequences of violence and aggression towards employees in the German healthcare and welfare system: a cross-sectional study. BMJ Open.

[58] Bojić M, Bole U, Bregar B (2016). Frequency and characteristics of patient violence against healthcare providers in emergency and psychiatric care settings. Slov Nurs Rev.

[59] Bregar B, Peterka Novak J, Možgan B (2011). Experiencing stress by psychiatric nurse practitioners. Slov Nurs Rev..

